# Radial head replacement versus reconstruction for the treatment of the terrible triad injury of the elbow: a systematic review and meta-analysis

**DOI:** 10.1007/s00402-019-03111-z

**Published:** 2019-01-17

**Authors:** Steven Kyriacou, Yash Gupta, Harraj Kaur Bains, Harvinder Pal Singh

**Affiliations:** Clinical Division of Orthopaedic Surgery, Department of Orthopaedic Surgery, University Hospitals Leicester, Leicester General Hospital, Gwendolen Road, Leicester, LE5 4PW UK

**Keywords:** Terrible, Triad, Radial, Head, Replacement, Reconstruction

## Abstract

**Introduction:**

The terrible triad injury of the elbow (TTIE) remains challenging to manage and has been associated with high complication rates and poor outcomes. There is a trend towards performing radial head replacement (REP) in preference to radial head reconstruction (REC) as arthroplasty provides early stability and may allow mobilisation sooner, potentially resulting in a better functional outcome. This systematic review compares the outcome of patients with TTIE treated with either REC or REP.

**Materials and methods:**

MEDLINE, Embase, and CINAHL were searched for studies published in English involving at least ten patients exclusively with a TTIE managed operatively, including both patients with either REC or REP. Data collection was in accordance with the Preferred Reporting Items for Systematic Review and Meta-analysis protocol. The outcomes of interest were Mayo Elbow Performance Score (MEPS) and range of motion (ROM). Post-operative complications were also compared.

**Results:**

9 studies involving 210 patients were included (98 REPs and 112 RECs). There was no statistically significant difference (*p* = 0.51) demonstrated between in the mean MEPS of the REP group (mean 88.6) and REC group (mean 88.5). Similarly, there was no statistically significant difference demonstrated between the REP and REC groups in terms of ROM. The risk of re-operation was high in both the REP (18.4%) and REC (17.9%) group. The overall complication rate of all patients included in the study was high (65%).

**Conclusions:**

Comparable results with good outcomes in terms of functional scores and ROM can be achieved with both REP and REC when treating TTIE, although the re-operation rate for both remains relatively high. Given there is no apparent clear advantage between the two treatment groups, we would suggest that REC should be performed when a satisfactory fixation can be achieved as the longevity of REP in young patients with a TTIE is currently uncertain.

## Introduction

A terrible triad injury of the elbow (TTIE) describes a dislocation of the elbow with associated fractures of the radial head and coronoid process of the ulna. The term ‘terrible triad’ in this context was originally coined by Hotchkiss in 1996 [1] in reference to the inherent difficulty in treating these injuries and their historically poor outcomes.

Stability within a normal elbow is conferred by the highly congruous nature of the joint and the interaction between the articular surfaces and soft-tissue stabilisers [2]. The combined loss of the postero-lateral stabilisation of the lateral ulnar collateral ligament (LUCL), the valgus buttress of the radial head, and the anterior buttress of the coronoid in TTIE result in a highly unstable elbow [3]. As a result of the unstable nature of this injury, conservative management is rarely a viable option with high failure rates and patients suffering from chronically unstable, painful, and stiff elbow. It is, therefore, widely accepted that the management of TTIE should be operative in the vast majority of cases [4].

An improved understanding of elbow biomechanics, advances in fixation techniques and implants, and the application of treatment algorithms and standardised surgical protocols have led to better outcomes being reported [2, 5–9].

The aim of operative management is to achieve a stable elbow which permits early rehabilitation, and to achieve this, each of the individual bony and soft-tissue components of the injury must be addressed in a sequential fashion, generally from deep to superficial [2, 7]. This includes fixation of the coronoid fracture, radial head fixation or replacement, repair of the LUCL, and in reserved cases in which there is ongoing instability following this, repair of the medial collateral ligament, and application of a hinged external fixator [10].

Whilst this protocol is currently widely accepted, the optimal technique by which to address each of these individual components continues to be open to debate. In particular, the decision to either fix or replace the radial head remains controversial. There has, however, been a recent trend observed towards an increase in the use of REP in radial head fractures, as arthroplasty provides early stability and may allow mobilisation sooner, potentially resulting in a better functional outcome [11, 12].

The aim of this systematic review was, therefore, to evaluate and compare the outcomes of patients who had undergone either radial head replacement (REP) or radial head reconstruction (REC) as a result of a TTIE based on current published literature and assess whether there is any difference in outcome between the two treatment groups.

## Methods

This systematic review was performed in accordance with the Preferred Reporting Items for Systematic Review and Meta-analysis (PRISMA) protocol. MEDLINE, Embase, and CINAHL were searched on the 23rd of May 2016 by a clinical librarian using combinations of the following search terms: “terribletriadinjury, elbow, elbowinjury, elbowinjuries, terribleANDtriadANDinjury”. The review was registered with the ‘University of York Centre for Reviews and Dissemination’. No prior review protocol was published.

A total of 165 studies were identified in the initial database search, within which there were no prospective, randomised studies. A further 6 studies were identified through the references listed in a published systematic review (1 of the 165 studies) which were not found in the initial database search. Recent and relevant systematic reviews were also searched to identify other potentially relevant studies. Studies were considered for inclusion in the review if they included more than ten participants with TTIE (i.e., elbow dislocation, radial head fracture, and coronoid process fracture), contained data which allowed comparison between REP and REC to be made and were published in English within the last 10 years. Studies were excluded from the review if they included participants with injuries other than TTIE or if they were published as letters, review articles, or case reports. Studies including patients under 16 years of age were also excluded as were studies in which some patients were managed with total elbow replacements.

**Fig. 1:**
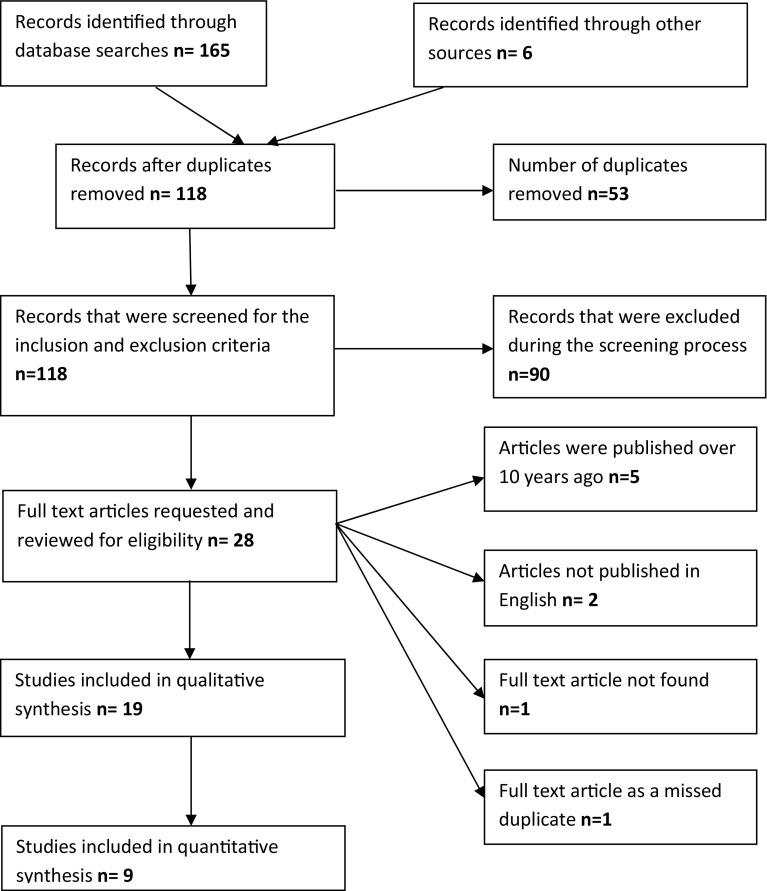
PRISMA flow chart

28 published articles underwent full text review and a further 9 were excluded. Of these, 5 were excluded, as they were published more than 10 years ago, 2 were excluded, because they were not printed in English, 1 was excluded, because the full article text could not be sourced, and 1 was excluded, because it was missed as a duplicate in the initial screening process. Of the 19 remaining articles, only 9 [2–4, 13–18] had sufficient data to make a quantitative comparison between REP and REC based upon the outcomes of interest (Fig. 1).

Data were extracted by two independent reviewers who consulted with a third reviewer to resolve any disagreements. The following data were extracted from each eligible study: author details, number, sex, mean age of patients, mean length of follow-up, Mason classification of radial head fracture, Regan–Morrey classification for coronoid fractures, treatment of radial head fracture, functional scores [Mayo elbow performance score (MEPS), Disabilities of the Arm, Shoulder, and Hand (DASH) scores, and American Shoulder and Elbow Surgeons (ASES) score], range of motion (ROM), and any post-operative complications. The primary functional outcome of interest was MEPS and secondary outcome was ROM. Post-operative complications were also explored and compared.

A meta-analysis was performed comparing both MEPS and ROM between patients with REP and REC in the nine studies. Data were incomplete for DASH scores, so meta-analysis was not performed. Differences were assessed using the *t* test and weighted mean differences with 95% confidence intervals using a random effects model. Review Manager (RevMan) (Version 5.3. Copenhagen: The Nordic Cochrane Centre, The Cochrane Collaboration 2014) was used to generate Forest plots for the outcomes of interest and measuring consistency of studies included within the meta-analyses.

## Results

The total number of participants in the nine included studies was 210 with 98 REP and 112 REC. 58% of the patients were male and 42% were female. Four studies [2, 4, 14, 18] provided individual data regarding gender distribution between the two cohorts. From these studies, 58% of patients were male in the REP cohort and 67% were male in the REC cohort. The age range for REP was 19–77 years and for REC was 22–81 years (Table 1). The mean age of the 210 patients included in the study was 41.3 years. The mean follow-up time was 39.7 months (range 9–97 months).

**Table 1:** Demographic characteristics of the studies included in the systematic review

Author, year published	Patients, number			Gender Male (female)			Mean age (range) Years			Mean length of follow-up (range), months	Radial head fracture classification ^A^	Coronoid fracture classification ^B^
	Radial head replacement (REP)	Radial head reconstruction (REC)	Overall	REP	REC	Overall	REP	REC	Overall			
Watters (2014)	30	9	39	NA	NA	21 (18)	NA	NA	48 (22–76)	24 (18–53)	NA	NA
Jeong (2010)	3	10	13	NA	NA	7 (6)	NA	NA	43.8 (15–76)	25 (18–41)	I–III	I–III^C^
Leigh (2012)	11	13	24	6 (5)	6 (7)	12 (12)	45.4 (19–67)	42.2 (29–56)	43.5	40.6	I–III	I–III
Pierrant (2015)	7	11	18	NA	NA	12 (6)	NA	NA	43.8 (19–56)	31.5	II–III	I–III
Toros (2011)	5	11	16	3 (2)	8 (3)	10 (5)	40.6 (34–50)	33 (24–46)	34 (24–50)	34.5 (14–110)	II–III	I–II
Chemama (2010)	4	10	14	3 (1)	9 (1)	12 (2)	39.5 (29–52)	40.4 (25–50)	NA	63 (15–128)	I–III	I–II
Giannicola (2015)	16	10	26	7 (9)	6 (4)	13 (13)	53.1 (35–77)	50.6 (26–81)	52 (26–81)	31 (12–87)	II–III	I–III
Yan (2015)	20	19	39	NA	NA	18(21)	36.5 (23–51)	35.5(22–48)	36 (22–51)	36	III	I–III
Zhang (2014)	2	19	21	NA	NA	17 (4)	NA	NA	38.4 (17–63)	32 (24–48)	I–III	NA

*NA* where data are unavailable/ not specified

^A^Radial Head–Mason classification

^B^Coronoid fracture based on Regan–Morrey classification

^C^O’driscoll classification

The implants used for REP varied between studies according to the surgeons’ preference and included both monobloc and modular prostheses. Methods of REC again varied between studies depending on surgeons’ preferences, but included the use of both screws and plates.

The indications to perform either REC or REP varied between included studies. Five of the included studies stated that REC was performed where technically possible [2, 13–16]. Watters et al. [3] stated that REC was performed when ‘there were fewer than four articular fragments; otherwise, it was replaced’. Although specific indications were not described by Giannicola et al. REC was performed in all, but 1 patient with a Mason II fracture and REP performed in all, but 2 patients with a Mason III fracture in their study [4]. Yan et al. [17] again did not describe specific indications, but all 39 patients included within their study had a Mason III fracture of the radial head, of which 20 underwent REP and 19 REC. With the exception of one of the nine studies which included 24 patients [2], the Mason grade of each of the remaining 186 patients was specified. Within the REC group, 52.5% of patients had Mason I or II fractures of the radial head and 47.5% had Mason III fractures. Within the REP group, 20.7% had Mason I or II fractures of the radial head and 79.3% had Mason III fractures. The greater proportion of patients with Mason III fractures in the REP group was found to be statistically significant when Fischer’s exact test was performed (*p* = 0.0001).

In all 9 studies included in this systematic review, the coronoid process fracture and LUCL injury were also managed operatively using various methods. For the repair of the coronoid process, seven studies [3, 4, 14–18] reported the use of sutures, five studies [2, 3, 13, 15, 17] reported the use of screws, and three studies [13, 16, 17] also included the use of plates. Eight studies [2–4, 13–15, 17, 18] reported repair of the LUCL with sutures, three studies included the use of suture anchors [2, 16, 17], and one study described repair of the LUCL via a bone tunnel [3].

### Patient outcome scores

**Fig. 2:**
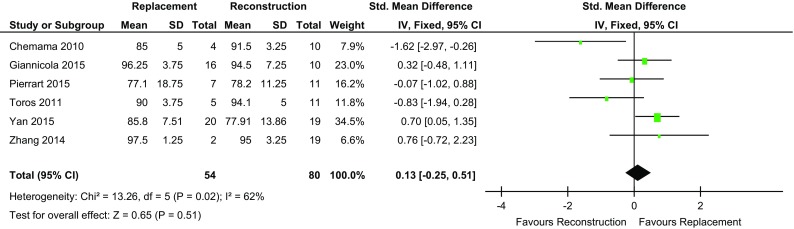
Forest plot MEPS

Three studies [14, 15, 18] found the mean MEPS to be greater in the REC group and three favoured REP [4, 13, 17]. Across the six studies which reported MEPS, the mean for the REP group was 88.6 (range 60–97) and 88.5 for REC group (range 55–95). There was no significant difference demonstrated between the mean MEPS of the REP cohort (*n* = 54) and the REC cohort (*n* = 80) (*p* = 0.51) (Table 2; Fig. 2).

**Table 2:** Patient outcome scores presented in the studies included in the systematic review

	Patients, number		MEPS Mean (SD)		ASES Mean (SD)			DASH Mean (SD)	
	Radial head replacement (REP)	Radial head reconstruction (REC)	REP	REC		REP	REC	REP	REC
Watters (2014)	30	9	NA	NA		NA	NA	16.1 (NA)	15.7 (NA)
Jeong (2010)	3	10	NA	NA		NA	NA	NA	NA
Leigh (2012)	11	13	NA	NA		89 (42.2)	81 (35.2)	10.8 (10.2)	9.2 (4.1)
Pierrant (2015)	7	11	77.1 (18.7)	78.2 (11.2)		NA	NA	NA	NA
Toros (2011)	5	11	90 (3.7)	94.1 (5)		NA	NA	11.8 (6.2)	7.9 (8)
Chemama (2010)	4	10	85 (5)	91.5 (3.2)		NA	NA	NA	NA
Giannicola (2015)	16	10	96.2 (3.7)	94.5 (7.2)		90.6 (43.1)	90.3 (44.7)	NA	NA
Zhang (2014)	2	19	97.5 (1.2)	95 (3.2)		NA	NA	NA	NA
Yan (2015)	20	19	85.8 (7.5)	77.9 (13.8)		NA	NA	NA	NA

*NA* not available

Three studies included data for DASH scores with a mean score of 12.9 in the REP cohort and 10.9 in the REC cohort. ASES score was available in only two of the included studies with a mean of 89.9 in the REP group and 85.7 in the REC group (Table 2).

### Range of motion

**Fig. 3:**
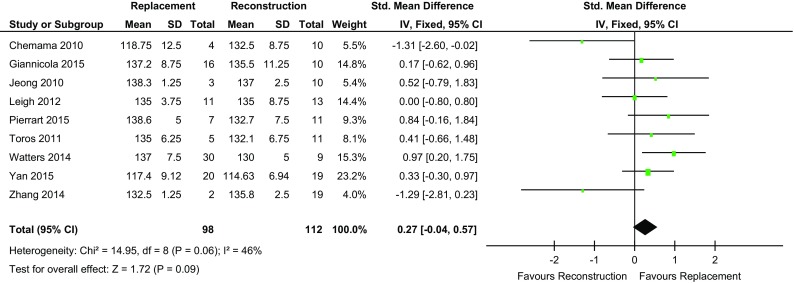
Forest plot flexion

Six of the studies [3, 4, 15–18] showed that the REP cohort had greater flexion compared to two studies for the REC cohort [13, 14]. The degree of flexion was the same in both cohorts in one study [2]. The mean flexion in the REP group was 132.2° (range 119–139) and 131.7 (range 130–136) in the REC group. Overall, there was no statistically significant difference between the two groups (*p* value = 0.09) (Fig. 3).

**Fig. 4:**
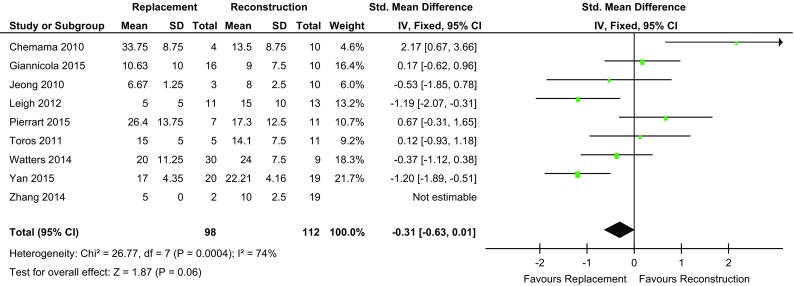
Forest plot extension

Four of the studies [4, 14, 15, 18] showed that the REP cohort had a greater maximum extension (fixed flexion deformity) compared to four studies for the REC cohort [2, 3, 16, 17]. The mean maximum extension in the REP group was 15.5° (range 5–34) and 28.29 (range 8–24) in the REC group. The difference between the two groups was not found to be statistically significant (*p* = 0.06) (Fig. 4).

**Fig. 5:**
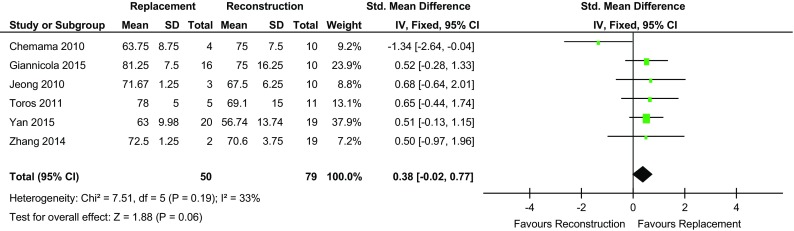
Forest plot pronation

Five of the studies [4, 13, 16–18] showed that the REP cohort had a greater maximum pronation compared to one study for the REC cohort [14]. The mean maximum pronation in the REP group was 71.7° (range 64–81) and 69.0° (range 67–75) in the REC group. The difference between the two groups was not found to be statistically significant (*p* = 0.06) (Fig. 5).

**Fig. 6:**
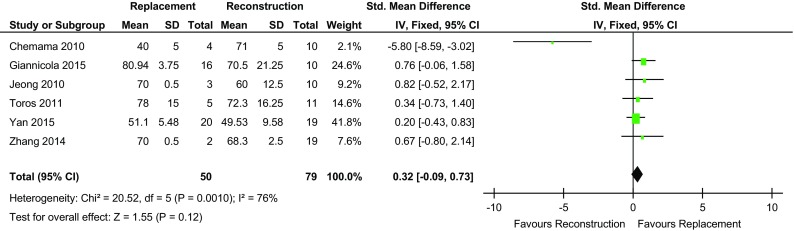
Forest plot supination

Five of the studies [4, 13, 16–18] showed that the REP cohort had a greater maximum supination compared to one study for the REC cohort [14]. The mean maximum supination in the REP group was 65.0° (range 40–70) and 65.3° (range 60–72) in the REC group. The difference between the two groups was not found to be statistically significant (*p* = 0.12) (Fig. 6).

### Complications

Table 3 summarises all complications reported in each of the studies included within this systematic review, including those which did and did not require re-operation.

**Table 3:** Summary of complications reported in the studies reported in the systematic review

Author, year published	Complications requiring reoperations		Complications not requiring reoperations		Unspecified complications
	Replacement	Reconstruction	Replacement	Reconstruction	
Watters (2014)	3 overstuffed prosthesis (implant revised) 4 contracture releases	1 failed fixation 2 residual instability 1 contracture releases	2 coronoid malunion	8 mild arthrosis 3 moderate arthrosis 9 coronoid malunion 1 failed fixation- failure of LUCL repair	0
Jeong (2010)	1 ulnar nerve release	0	0	2 heterotrophic ossification	0
Leigh (2012)	1 joint subluxation (implant revised) 1 deep infection	2 symptomatic non-union 1 migration of a threaded Kirschner wire 2 unable to gain functional movement	0	0	2 heterotopic ossification
Pierrart (2015)	NA	NA	NA	NA	2 wound dehiscence 1 dislocation of the humeroulnar joint 2 radial head prosthesis dislocations 1 radial head non-union 6 coronoid non-union 1 persistent dislocation 4 moderate arthritis 5 mild arthritis 11 heterotopic bone formation around elbow joint
Toros (2011)	NA	NA	NA	NA	6 grade 1 arthritis (Broberg Morrey) 4 ulnar neuropathy
Chemama (2010)	1 severe pain on the lateral column (implant revised) 1 ulnocarpal impingement	1 instability	1 osteoarthritis	0	0
Giannicola (2015)	2 open debridement due to stiffness 2 late FFS loosening and olecranon bursitis	2 open debridement due to stiffness	2 elbow instability 3 heterotopic ossification 1 sensory ulnar nerve neuropathy	1 stiffness 1 instability 1 coronoid malunion 1 radial head malunion 1 coronoid non-union 2 heterotopic ossification	0
Zhang (2014)	0	1 infection needing surgical debridement and antibiotics	0	1 ulnar neuropathy 1 radial non-union 2 heterotopic ossification	8 grade 1 arthritis (Broberg Morrey)
Yan (2015)	1 stiffness due to overstuffing (implant revised) 1 Heterotopic ossification	4 stiffness 2 Heterotopic ossification 1 failure of fixation radial head	1 displaced coronoid fragment	2 displaced coronoid fragment	0

*NA* data unavailable/not specified

The number of complications requiring re-operation in the REP group was 18 out of 98 cases (18.4%) and the number of complications requiring re-operation in the REC group was 20/112 (17.9%). The difference in re-operation rates between the two groups was not found to be statistically significant (*p* = 1.00). The overall number of complications which required re-operation in all patients included in the study was 38 out of 210 (18.1%). The overall complication rate of all patients included in the study was 137 out of 210 (65%).

As would be expected, there were differences in the indications for re-operation between the two groups. In the REP group, there were six cases, where the implant was revised due to incorrect sizing and a further two cases of reported radial head prosthesis dislocations, though it was not confirmed within this paper [15] if these too were also revised. In the REC group, there are two reported cases of failed fixation and two cases of symptomatic non-union which required revision surgery.

The most commonly reported complications overall which did not require re-operation were arthrosis (35 cases) and heterotopic ossification (22 cases).

## Discussion

The radial head plays a critical role in the stability of the elbow and is an important secondary stabiliser against valgus loading and posterior translation [10, 19]. As a result, excision of the radial head is contraindicated in TTIE due to the high risk of associated complications including persistent instability, arthrosis and proximal migration of the radius [5, 17, 20]. Therefore, to restore radial head stability and minimise the risk of these complications when treating patients with TTIE, either REC or REP is recommended in addition to repair of the coronoid fracture and LUCL [5–7, 10]. However, the issue of whether either of these methods results in superior outcomes compared to the other at present remains largely unanswered and there are currently relative few published studies which directly address this question.

Leigh et al. [2] in their retrospective study of 23 patients comparing REC versus REP in the treatment of TTIE concluded that ‘comparable results can be obtained with repair or replacement of the radial head in this injury pattern in the short term’, but recommended REC ‘especially in younger patients’ despite identifying a higher re-operation rate in REC compared to REP. Similarly, Watters et al. [3] in their retrospective study of 39 patients identified no difference between the REP and REC group in terms of ROM and elbow scores. They did, however, find a statistically significant difference between the two groups in terms of post-operative instability, with 3/9 patients in the REC group having issues with instability compared with no patients in the REP group (*p* = 0.0009). On the basis on this finding, the authors concluded that REP affords ‘the ability to obtain elbow stability with comparable overall outcomes when compared to ORIF’. The retrospective study by Yan et al. [17] of 39 patients (all of whom had Mason III radial head fractures) found that both ROM and elbow scores were statistically superior in the REP group compared to the REC group and, therefore, concluded that REP ‘might be a more effective approach to better managing a terrible triad of the elbow’ compared to REC.

This systematic review and meta-analysis found that there was no statistically significant difference in MEPS between TTIE managed with either REP or REC, with a generally good result being achievable irrespective of which method was used (mean MEPS 88.6 versus 88.5, respectively). In terms of ROM, no statistically significant difference was demonstrated between the two groups in terms of flexion, extension, pronation, and supination. There was no statistically significant difference found between the re-operation rate in the REP group and the REC group (18.4% versus 17.9%, respectively), though there were differences in the indications, as would be expected, with six patients in the REP group needing revision due to incorrect sizing and four cases in the REC group needing revision due to failure of fixation/symptomatic non-union. The overall complication rate for all patients with TTIE managed operatively was, however, found to be high at 65%. Despite this, surgical intervention remains warranted, as these injuries have previous been demonstrated to have very poor outcomes with a propensity for pain, recurrent instability, and stiffness when managed conservatively [21].

There are issues specific to both treatment modalities. As observed in this study, ‘overstuffing’ can be a potential problem and correctly sizing the radial head prosthesis can be difficult with an associated learning curve in avoiding this complication. Overstuffing the radio-capitellar joint by as little as 2.5 mm has been demonstrated to significantly alter kinematics and joint pressures which can result in pain and stiffness [22]. The use of a modular prosthesis is, therefore, preferable, as it allows head and stem diameters and heights to be independently adjusted to achieve an optimal fit [2]. In instances, where REC is performed, an insecure fixation must be avoided, as the stresses across the radial head during the post-operative period may result in subsequent failure of fixation [2] and results may potentially be less favourable with comminuted radial head fractures involving three or more fragments [23].

There are weaknesses associated with this study. Data from 210 patients were included and a greater number would help increase the significance of the findings. A mean follow-up of 39.7 months entails any differences in outcome between the two groups which may potentially occur beyond this point will not have been assessed. It, therefore, does not provide sufficient time to observe late complications such as implant loosening which may cause deterioration in ROM and MEPS and potentially result in revision surgery. Eight of the nine papers included within the systematic review were retrospective with the deficiencies associated with studies of this nature.

As a result of the multi-faceted approach in treating TTIE operatively, there is inherently a large degree of heterogeneity in the data being analysed due to additional differences in the methods of treatment between the various studies other than the choice between REP and REC. These differences include the surgical approach utilised and method of fixation of the coronoid fracture and LUCL. These factors may all also potentially influence the observed outcomes and, therefore, make an exact, matched comparison hard to achieve.

We found a statistically significant (*p* = 0.0001) higher proportion of patients with Mason III fractures in the REP group than in the REC group. Therefore, the results observed in this study may be influenced by the fact the group of patients receiving both REC and REP although similar, may be subtly different due to there being a greater percentage of patients who initially sustained higher energy injuries with more comminution in the REP group, thereby compromising comparison between the two treatment groups.

**Fig. 7:**
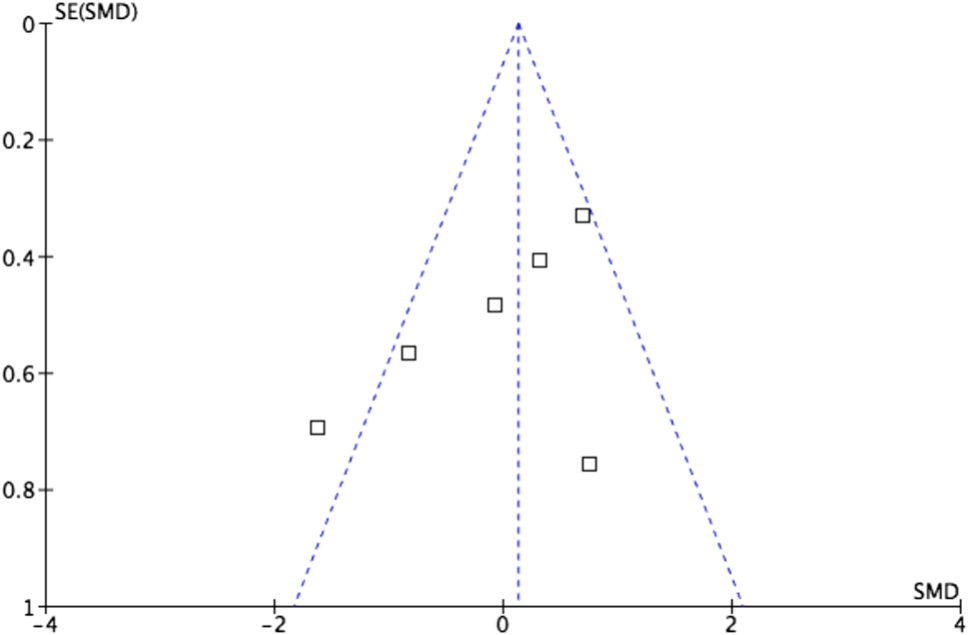
Funnel plot demonstrating mild heterogeneity of MEPS between studies included in meta-analysis

There is a risk of bias potentially affecting the evidence as a result of the complete absence of any available randomised trials that could be included within the study. There is also slight heterogeneity between studies as one of the studies lies outside the funnel plot for MEPS score (Fig. 7).

Despite these weaknesses, we feel that there is sufficient evidence from this study to conclude that no clear advantage has been demonstrated between the two treatment modalities in the management of TTIE in the literature, thus far. It, therefore, seems reasonable to advocate treating each case on its individual merits, as both methods have been demonstrated to provide good outcomes in the short term. However, given the current lack of long-term evidence on the longevity of REP for TTIE in what is in general a relatively young patient population (mean age 41.3 years in this study), we would suggest that REC should be performed in instances, where a satisfactory fixation is technically achievable. Further prospective, matched, randomised controlled trials as well as studies with longer follow-up are required to allow a better comparison between REP and REC in the management of patients with TTIE to be made and to clarify the specific indications for their use.

## Conclusion

Comparable results with good outcomes in terms of functional scores and ROM can be achieved with both REP and REC, although the re-operation rate for both remains relatively high. Larger prospective, randomised studies are required to determine any differences in long-term outcomes between REP and REC in the treatment of patients with TTIE.
